# Increased risk of eczema after joint replacement: A population-based retrospective cohort study

**DOI:** 10.1097/MD.0000000000017914

**Published:** 2019-11-11

**Authors:** Po-Yuan Wu, Chih-Hsin Muo, Chun-Hao Tsai

**Affiliations:** a Department of Dermatology, China Medical University Hospital; b School of Medicine, China Medical University; c Management Office for Health Data; d Department of Orthopedics, China Medical University Hospital; e Department of Sports Medicine/School of Medicine, China Medical University, Taichung, Taiwan.

**Keywords:** arthroplasty, dermatitis, eczema, metal hypersensitivity

## Abstract

There are very few reports of eczema and other prosthetic-related allergic skin complications following arthroplasty. We aimed to assess the risk of eczema after joint replacement.

We performed a retrospective population-based cohort study in 2024 joint replacement patients using the Longitudinal Health Insurance Database. For comparison, 8096 controls were selected, with 4 control subjects for each joint replacement patient matched for age, sex, and index year, to assess eczema risk. We examined 14-year cumulative eczema incidence associated with age, sex, immunity, disease history, and joint replacement location.

Eczema rates in the joint replacement patients were 38% higher than in the control group (57.90 vs 41.84 per 1000 person-years, respectively). Compared with the control group, joint replacement patients showed a 1.35-fold increased risk of eczema according to the multivariable Cox model (95% Confidence interval [CI] = 1.23–1.49). Knee replacement patients had higher eczema risk compared with the control group (Hazard ratio [HR] = 1.45, 95% CI = 1.33–1.70). Stratified by study period, the joint replacement cohort had a higher eczema risk after the 3-month follow-up.

Our study revealed that joint arthroplasty increased risk of eczema in this 14-year follow-up study, and this was not related to personal atopic history or gender.

## 1 Introduction

Artificial joint arthroplasty has excellent clinical results for the treatment of end-stage arthritis. Joint prosthesis composite materials are foreign to the body, and include plastic polymers, bone cement (methyl methacrylate), and metals, such as chromium, cobalt, and titanium alloys. Since the first reported case of allergy after arthroplasty in 1966,^[1]^ hypersensitivity reactions following orthopedic prostheses have increased in prominence in recent decades.^[2–7]^ Hypersensitivity to prostheses presents various symptoms, including skin reactions, chronic pain, loss of joint function, prosthesis loosening, and even periprosthetic fibrosis.^[8–12]^ Skin reactions to prostheses include contact dermatitis, vasculitis, urticaria, and erythema. Among these reactions, eczema is the most-reported hypersensitivity reaction observed after arthroplasty and may be associated with nickel, chromium, or cobalt allergies, and non-metals, such as silicon and bone cement components.^[13–15]^ However, the risk of eczema after joint replacement has only been reported in case reports, small patient cohorts, or meta-analyses.^[11,15–26]^ Unfortunately, there has only been 1 study reporting on metal allergy in knee arthroplasty, which was conducted in a Danish population.^[27]^ This suggests a need for nationwide population-based studies with long-term follow-up periods to provide a comprehensive overview of the overall incidence and risk factors. Therefore, the aim of this study was to evaluate whether joint arthroplasty is associated with an increased risk of eczema and to identify the medical or demographic risk factors over a 14-year follow-up period.

## 2 Methods

### 2.1 Data source

This was a retrospective cohort study using the Longitudinal Health Insurance Database (LHID), which contains 1 million beneficiaries randomly selected from the Taiwan National Health Insurance Programme in 2000. This program is a compulsory insurance program and the LHID includes all de-identified data, including medial claims and treatments as well as both outpatient and inpatient visits, for each beneficiary from 1996 to 2013. The China Medical University and Hospital Institutional Review Board approved this study. To define diseases and treatments from the LHID, we used the International Classification of Diseases, Ninth Revision, Clinical Modification (ICD-9-CM) and ICD-9-CM operation codes.

### 2.2 Study population

Patients with joint replacements (ICD-9-CM operation code 81.5, 81.73, 81.80, 81.81, 81.84, and 81.97) following the Taiwan Ministry of Health and Welfare guidelines from 2000 to 2010 were selected (N = 8277). The date of joint replacement was defined as the index date. The exclusion criteria were as follows:

(1) Joint or revision replacement (ICD-9-CM operation code 81.53, 81.55, 81.59, and 81.97) history
(2) History of eczema (ICD-9-CM 690-692, and 698.3)
(3) Spine- or fracture-associated operation (ICD-9-CM operation code 79 and 81)
(4) Cellulitis (ICD-9-CM 682.5-682.9)
(5) Osteomyelitis (ICD-9-CM 730)
(6) History of Pyogenic arthritis (ICD-9-CM 711)
(7) History of end-stage renal disease (ICD-9-CM 585.6)

**Figure 1:**
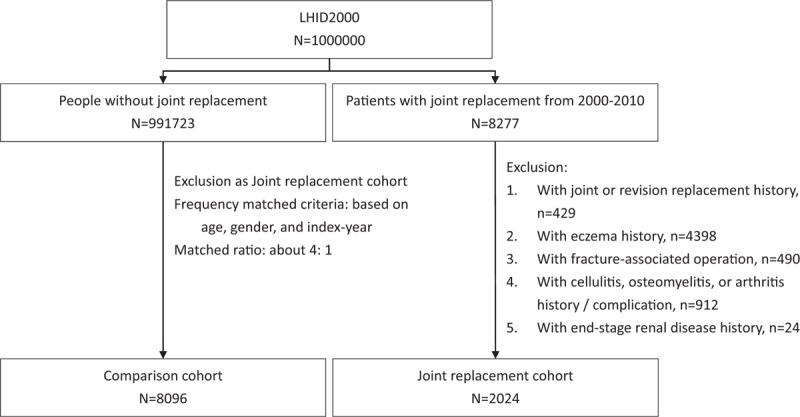
Flow chart of the study design and patient selection.

The control group was comprised of patients without any joint replacements listed in the LHID. The exclusion criteria were identical for the control group. Approximately 4 controls for each joint replacement patient were randomly selected, according to age (5-year stratum: for example, 0–4, 5–9, and 10–14), sex, and index year. Figure 1 presents the subject selection details.

### 2.3 Outcomes and baseline comorbidity

All study subjects were followed from the index date until the development of eczema, as diagnosed by a dermatologist on 3 unique visits. Those who did not develop eczema were followed until the end date of 2013 or until they withdrew from the program, whichever came first. The baseline comorbidities considered in this study included asthma (ICD-9-CM 493), allergic conjunctivitis (ICD-9-CM 372.05, 372.10, and 372.14), allergic rhinitis (ICD-9-CM 477), and immunity disorders including lupus erythematosus (ICD-9-CM 710.0) and rheumatoid arthritis (ICD-9-CM 714.0).

### 2.4 Ethical considerations

The scientific committee of the China Medical University Hospital and the ethical committee in the China Medical University Hospital waived approval for the human protocol for this investigation and each author certifies that all investigations conformed with ethical principles of research. This work was performed at the China Medical University Hospital, Taichung, Taiwan.

### 2.5 Statistical analysis

A Chi-Squared test was used to determine differences among age (<50, 50–64, and 65+ years), sex, and comorbidities between joint replacement and control cohorts. The variables are presented as number of cases and as a percentage of the total sample. Student *t* test was used to test differences in mean ages between the 2 cohorts. The results are shown as the mean and standard deviation. The rate per 1000 person-years was counted as the sum of eczema development divided by the sum of person-years during the study period. We used a Cox proportional hazard regression analysis to estimate the hazard ratio (HR) and compared the 95% confidence interval (CI) for eczema between joint replacement and control cohorts. A multivariable Cox model was adjusted for age, sex, and comorbidity. For sensitivity analysis, the age-, sex-, and comorbidity-stratified analyses were assessed. The association between developing eczema and the location of the joint replacement was estimated. We also estimated the combined effect for eczema between joint replacement and comorbidity. Because this study violated the Cox proportional hazard assumption via a scaled Schoenfeld residuals test (*P* = .03), we analyzed the association between eczema and joint replacement stratified by follow-up time. To plot the cumulative incidence in the 2 cohorts, we used a Kaplan–Meier analysis and a log-rank test to test the difference between the 2 cohorts. All the analyses were performed with SAS 9.4 software (SAS Institute Inc., Cary, NC, USA) and all statistical tests were two-sided. A *P* value of <.05 was considered statistically significant. We used SPSS V18 software (IBM Corp., Armonk, NY, USA) to plot the cumulative incidence.

## 3 Results

In total, 2024 joint replacement patients and 8096 control patients were selected for this study. There were no significant differences in age or sex between the joint replacement group and the age- and sex-matched controls. The mean age was 66.4 ± 12.1, with more women (63.6%) than men (36.4%) in the joint replacement cohort (Table 1). Compared to the control group, joint replacement patients were likely to have more comorbidities, including asthma (10.4% vs 7.5%), allergic conjunctivitis (22.5% vs 19.8%), and immunity disorders (1.3% vs 0.3%).

**Table 1:**
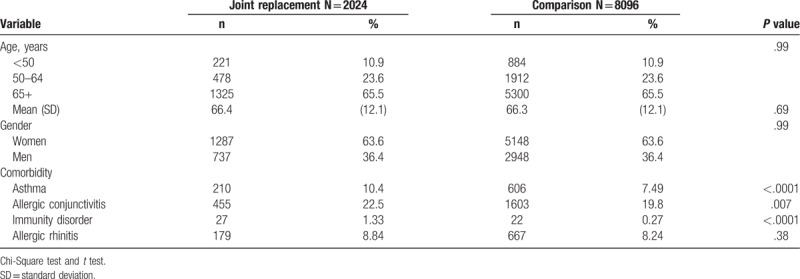
Demographics and comorbidity between patients with and without joint replacement.

**Figure 2:**
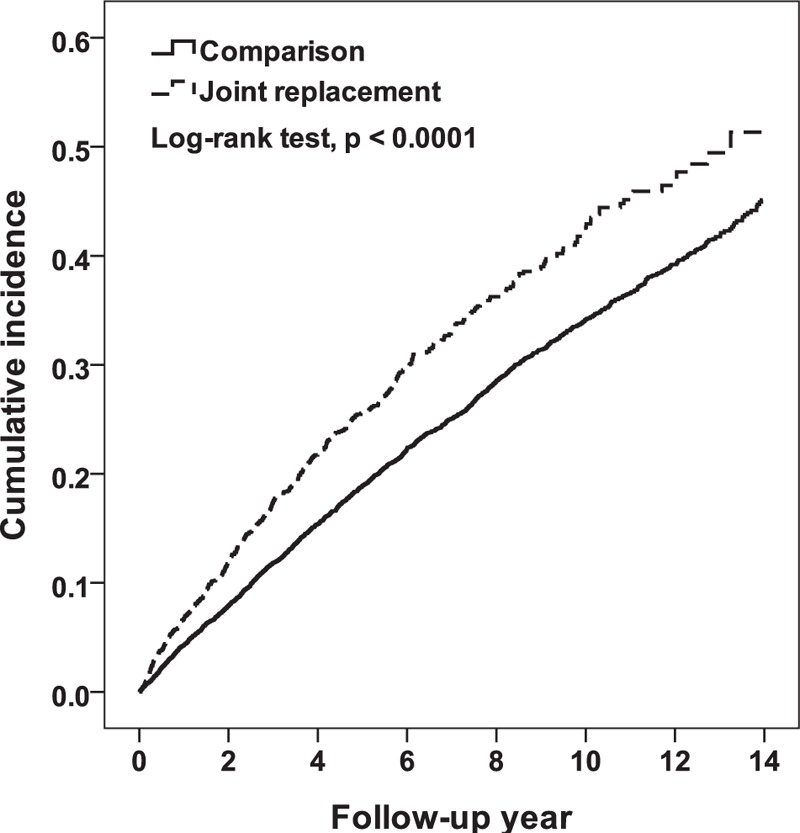
The cumulative incidence of eczema in the joint replacement patients was 6.21% higher than that in the control group after the 14-year follow-up period (log-rank, *P* < .001).

During the study period, 511 and 2162 patients developed eczema in the joint replacement and control cohorts, respectively, with rates of 57.90 and 41.84 per 1000 person-years, respectively (Table 2). From the Kaplan–Meier analysis, the cumulative incidence in joint replacement patients was 6.21% higher than in the control after the 14-year follow-up (log-rank *P* < .001) (Fig. 2). Compared with controls, joint replacement patients had 1.38- and 1.35-fold greater eczema risk in the crude and multivariable Cox models, respectively (95% CI = 1.25–1.52, and 1.23–1.49, Table 2). In the age-, sex-, and comorbidity-stratified analyses, the joint replacement cohort still presented with a higher incidence of eczema than the control cohort. However, in patients younger than 50 years of age, the difference was not statistically significant.

**Table 2:**
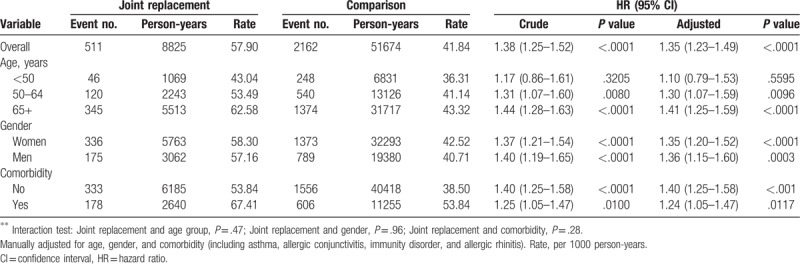
Incidence and hazard ratio for eczema in joint replacement patients compared with comparisons in Cox proportional model stratified by age, gender, and comorbidity.

Table 3 presents the association between eczema and the location of the joint replaced. Lower limb replacement was classified as hip and knee replacement. The eczema incidence was the highest in upper limb replacement patients (79.95 per 1000 person-years), followed by lower limb replacement patients and controls (57.84 and 41.84 per 1000 person-years, respectively). In the multivariate Cox model, only lower limb replacement patients had a significantly higher eczema risk compared with control patients (HR = 1.35, 95% CI = 1.23–1.49). There was no statistically significant difference when comparing patients with upper limb replacements because of the small number of patients. Compared with the control group, patients with hip or knee replacement had significantly higher eczema risk (HR = 1.24 in the hip replacement group and 1.45 in the knee replacement group, 95% CI = 1.08–1.43 and 1.28–1.64, respectively).

**Table 3:**
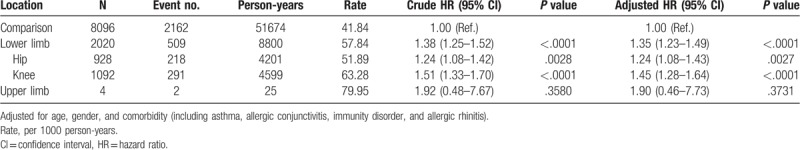
Incidence and hazard ratio for eczema among joint replacement location in Cox proportional model.

Table 4 shows the combined effect for eczema, i.e. between joint replacement and comorbidity, according to an age- and sex-adjusted Cox model. Compared to the control group, patients with only joint replacements had a 1.29-fold greater risk of eczema (95% CI = 1.15–1.45). The eczema risk increased in joint replacement patients, with the comorbidity number increasing from 1.29 for joint replacement patients without comorbidities (95% CI = 1.15–1.45) to 1.56 for joint replacement patients with any 1 comorbidity (95% CI = 1.32–1.84), and up to 1.74 for joint replacement patients with ≥2 comorbidities (95% CI = 1.17–2.61).

**Table 4:**
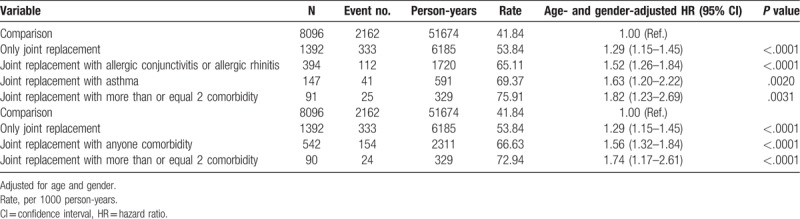
Joint effect for eczema between joint replacement and comorbidity in Cox proportional model.

In the study period-stratified analysis, we grouped patients from the study period into 4 groups: ≤1 month, 2 to 3 months, 4 to 12 months, and >12 months (Table 5). After 1 month of follow-up, the joint replacement cohort had significantly higher eczema risk. The highest eczema risk in joint replacement patients compared with the control group was in the 2 to 3 month study period (HR = 2.71, 95% CI = 1.71–4.29).

**Table 5:**
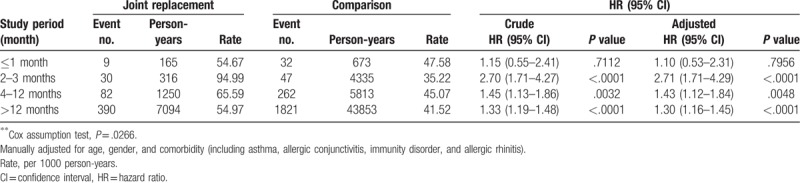
Incidence and hazard ratio for eczema in joint replacement patients compared with comparisons in Cox proportional model stratified by study period.

## 4 Discussion

Eczema can occur following implantation in orthopedic prosthesis and has been associated with nickel, chromium, or cobalt composites in the implants.^[7,13,28]^ The symptoms of skin lesion after joint replacement can be classified as

1. allergic contact dermatitis from metals which are components of prosthesis,^[29]^ this type the eczema rash mostly located near the prosthesis area and
2. systemic contact dermatitis or symmetrical drug-related intertriginous and flexural exanthema (SDRIFE).^[30]^

Of these materials, chromium is considered most likely to cause dermatitis after joint arthroplasty.^[27,31–33]^ A major difficulty in understanding skin conditions following implant is the lack of a universally feasible testing method, such as skin patch test or lymphocytes transformation testing (LTT), that results in underreporting. Another difficulty is the paucity of clinical studies providing clear data on a connection between metal sensitivity and implant outcomes.^[34,35]^ Our study revealed that eczema incidence rate was 57.90 per 1000 person-years and the adjusted overall HR was 1.35 after a 14-year follow-up. Our results are consistent with other reports in the literature; a systemic review revealed that the prevalence of metal allergy was higher after joint arthroplasty compared with the control group (odds ratio [OR] 1.52 (95% confidence interval [CI], 1.06–2.31)).^[25]^

The distribution of our study group demography is the same as in previous literature, with more women than men and most patients aged over 65 years.^[36]^ We observed a higher percentage of allergic and immunity disorders in the joint replacement group in our study. Men and women had approximately the same increase in risk of developing eczema after joint replacement. The result did not meet our expectations because the overall prevalence of metal hypersensitivity in the general population is estimated to be between 10% and 15% and is higher in women than in men.^[5,37]^ Our study also showed that age influenced the risk of eczema. In a study of 493 trauma patients with an average age of 39 years, Swiontkowski et al found that the prevalence of metal sensitivity was considerably lower than the rates reported in the general population, leading the authors to suggest that metal sensitivity may be less prevalent in younger populations.^[38]^

After further stratifying the risk of eczema after arthroplasty by anatomical site, there was a relatively higher risk in the hip and knee arthroplasty group than in the control group compared with the upper limb comparison, likely due to the relatively small sample size for upper limb replacement (n = 4) in our study. Although upper limb joints are not weight bearing, abundant metal particles in macrophages in some tissue from resected, failed reverse total shoulder arthroplasties have been previously described.^[39]^ Further research is needed to isolate eczema risk in upper limb arthroplasty.

Duration since join replacement plays a role in the relationship between artificial prostheses and the development of eczema. Our study observed an increased eczema risk after 2–3 months, when the healing process is expected to finish. This increased risk of eczema after a period of time comes from clinical observations in the literature.^[10]^ Our results also indicate that eczema risk increased with time. Over time, prosthesis wears with use and the resulting particles accumulate around the joint, thereby activating the host immune response. The immune system responds to implant debris by forming myeloid progenitor cells and lymphoid stem cells, which are responsible for innate (non-specific) and adaptive (specific) immune reactivity, respectively. Cell-mediated delay type hypersensitivity with TH1 cells has been studied in the literature.^[40,41]^ The interplay between the resulting chemokine and cytokine expression and subsequent activation of innate and adaptive immunity is partially understood, but is limited due to a lack of basic understanding of a few central chemokines, including MCP-1, IL-8, and MIP-1.^[42]^

Due to increasing awareness of eczema risk after joint replacement, many predictive methods have been developed, such as the patch test and lymphocyte transformation test. These are important methods in diagnosing and evaluating implant allergies. However, the current viewpoint of these tests is that there is no association between post-operative allergic symptoms.^[41,43–45]^ The impact of pre-existing metal sensitivity on clinical outcomes, as demonstrated by preoperative history or patch testing, remains controversial.^[44,46]^ The reason that these methods cannot accurately predict allergic reactions may be explained in part by these reactions, which do not associate allergies with a single material or alloy, but rather a combination of innate and acquired immunity responses. Physicians should be aware of skin lesion complaints following joint replacement, even years after surgery. Self-reported skin allergies are an important first step in diagnosing and evaluating implant allergies.^[24]^ Eczema reactions do not necessarily indicate poor functional outcomes that require surgical revision.^[27,45]^ However, revision surgery is suggested if skin reactions lead to recurrent erythematous swelling and poor wound healing.^[47]^ Several studies have reported that the eczema condition has been resolved after revision to prostheses with ceramic-based components^[11,17,18,20–23]^ and uncemented prostheses.^[15,26]^

Our study had several limitations. Firstly, we relied on National Health Insurance Research Databases ICD 9 code to reach the diagnosis of eczema. No eczema location information in ICD 9 compared with ICD 10. Besides, the etiology of eczema is multiple included asteatotic, atopic, venous insufficiency, or contact-induced. Consequently, the incidence rate of contact dermatitis may be overestimated. This study revealed the HR is reliable as both groups exhibited the same eczema etiology and well-controlled for comorbidity. Secondly, information regarding arthroplasty prior to the year 2000 was unavailable, and thus, may have been misclassified in both cohorts. Third, joint replacement may be linked with osteoporosis due to steroid treatment of allergic or immune-related conditions which were associated with joint replacement in the study and could be, in turn, associated by themselves with eczema. As the result, it can partially explain in our study, joint replacement patients were likely to have more comorbidities including asthma, allergic conjunctivitis, and immunity disorders. Due to treatment effect of steroid for eczema, the hazard ratio may be underestimated. However, the main advantage of this study is the use of population-based data, which is highly representative of the general population.

## 5 Conclusion

Our data indicate that physicians may notice eczema risk long after joint replacement surgery. Although the immune mechanism of eczema following joint replacement is complex and not well understood, the trend among surgeons is to choose a hypoallergenic prosthesis or a biological joint preserving procedure in addition to developing novel allergy tests for select patients. Communication and collaboration between surgeons and dermatologists can identify the risk of eczema, as well as possible implant complications, in patients following joint arthroplasty.

## Acknowledgments

This study was supported in part by This study is supported in part by Taiwan Ministry of Health and Welfare Clinical Trial Center (MOHW108-TDU-B-212-133004), China Medical University Hospital (DMR-107-071/CRS-108-035), Academia Sinica Stroke Biosignature Project (BM10701010021), MOST Clinical Trial Consortium for Stroke (MOST 107-2321 -B-039 -004-), Tseng-Lien Lin Foundation, Taichung, Taiwan, and Katsuzo and Kiyo Aoshima Memorial Funds, Japan. We would like to thank Uni-edit (www.uni-edit.net) for editing and proofreading this manuscript.

## Author contributions

**Conceptualization:** Po-Yuan Wu, Chun-Hao Tsai.

**Data curation:** Chih-Hsin Muo, Chun-Hao Tsai.

**Formal analysis:** Chih-Hsin Muo, Chun-Hao Tsai.

**Investigation:** Chun-Hao Tsai.

**Methodology:** Chih-Hsin Muo, Chun-Hao Tsai.

**Resources:** Chun-Hao Tsai.

**Software:** Chih-Hsin Muo.

**Supervision:** Chun-Hao Tsai.

**Validation:** Po-Yuan Wu, Chun-Hao Tsai.

**Visualization:** Po-Yuan Wu.

**Writing – original draft:** Po-Yuan Wu, Chih-Hsin Muo.

**Writing – review & editing:** Po-Yuan Wu, Chun-Hao Tsai.
